# Response to Merz

**DOI:** 10.1186/s13063-023-07693-3

**Published:** 2023-10-06

**Authors:** Pepijn Al, Jamie Brehaut, Charles Weijer

**Affiliations:** 1 Rotman Institute of Philosophy, Western University, London, ON Canada; 2 Centre for Implementation Research, Clinical Epidemiology Program, Ottawa Hospital Research Institute, Ottawa, ON Canada; 3 School of Epidemiology and Public Health, University of Ottawa, Ottawa, ON Canada; 4 Departments of Medicine, Epidemiology & Biostatistics, and Philosophy, Western University, London, ON Canada

## Abstract

Jon Merz raises two objections to our article on the ethics of behavioral influences in trial recruitment. In this response, we defend our article against these objections. We argue that Merz’s critique rests on a misunderstanding of our article, defend the daily life standard as a guardrail for leveraging cognitive biases, and argue that rejecting all behavioral influences is not a helpful nor a sustainable answer to their increasing use in trial recruitment.

Jon Merz raises two objections to our article on the ethics of behavioral influences in trial recruitment [1]. Firstly, he argues that our argument rests on the incorrect assumption that consenting to research participation is the right choice for potential participants. Secondly, Merz objects to our proposal of using a daily life standard of behavioral influences, which states that “behavioral influence strategies could be considered prima facie autonomy-respecting if it is comparable to other uncontroversial behavioral influence strategies to which an individual is routinely exposed in their daily life [2].” Merz argues that this will devolve research ethics into “an ‘ethics’ of the marketplace” with advertisements based on the tools of propaganda [1].

We believe that Merz’s criticisms are based on a misunderstanding of our article as defending the “manipulation of the methods of recruitment” [1]. Our paper is a call for urgently needed guidance on how techniques that can be used to influence behavior should — and should not — be used, not a defense of “manipulative methods.” The use of behavioral influences is already widespread in clinical trials, as trialists seek to recruit participants in sufficient numbers. This often happens without explicit consideration by trialists and research ethics committees. Since behavioral influences can be used in unethical ways, ethical guidance is necessary to protect potential research participants and preserve public trust in clinical trials.

To illustrate both the promise and the ethical perils of behavioral influences, we analyzed two examples of behavioral influences, one leveraging cognitive biases, and the other patients’ trust in their physicians. Our analysis surfaces ethical issues in each example and gestures towards existing guidelines and potential additional guardrails. To be clear though, “we believe additional resources and guidance are needed. We have proposed [...] a few components of this guidance, but recognize that much more work is needed [2].”

Merz objects particularly to the use of “the daily life standard” as a potential guardrail. The daily life standard challenges the presumption that leveraging cognitive biases necessarily undermines people’s autonomy. It prompts research ethics committees to ask the question: if we consider people to be capable of autonomous decision-making in the presence of this influence in daily life, why should we consider the same influence to be autonomy undermining in research recruitment? But asking the question is a far cry from endorsing an “ethics of the marketplace.” We expect that some behavioral influences that leverage cognitive biases substantially undermine autonomy regardless of the setting and should not be used; others may be acceptable in the marketplace but exert an invidious effect in research; and yet others are innocuous with respect to autonomy in both settings. Additional ethical analysis and guidance is needed to assist researchers and research ethics committees in this determination.

Far from assuming that “research participation is the correct normative choice for potential subjects and that researchers (and ethics review boards) are therefore justified in purposefully using psychological manipulation to influence potential subjects’ decisions” [1], we argue that it is the quality of trial participation that matters. To improve on the quality of trial participation, we recommend that trialists who use behavioral influences should collect data on the experience of participants. We posit that regret is an important marker of an enrollment decision that was* not the right one for the participant*. Trialists should attend carefully to recruitment and other aspects of the conduct of the trial to “ensure (as much as possible) that their research participants do not regret their decision to enroll [2].”

Behavioral influences are already routinely and increasingly used in recruitment, sometimes in ethically permissible ways, sometimes in ethically impermissible ways. Rejecting all behavioral influences is not a helpful nor a sustainable answer to this trend. Instead, ethicists should help trialists and research ethics committees to distinguish between the permissible and impermissible types and usages of behavioral influences. This requires more research on specific influences, instead of rejecting their usage from the start.

## Data Availability

Not applicable.
